# Comparison of machine learning approaches for near-fall-detection with motion sensors

**DOI:** 10.3389/fdgth.2023.1223845

**Published:** 2023-07-26

**Authors:** Sandra Hellmers, Elias Krey, Arber Gashi, Jessica Koschate, Laura Schmidt, Tim Stuckenschneider, Andreas Hein, Tania Zieschang

**Affiliations:** ^1^ Assistance Systems and Medical Device Technology, Department for Health Services Research, Carl von Ossietzky University, Oldenburg, Germany; ^2^ Geriatric Medicine, Department for Health Services Research, Carl von Ossietzky University, Oldenburg, Germany

**Keywords:** near-fall, perturbation, CNN, machine learning, IMU, fall risk, mobile health

## Abstract

**Introduction:**

Falls are one of the most common causes of emergency hospital visits in older people. Early recognition of an increased fall risk, which can be indicated by the occurrence of near-falls, is important to initiate interventions.

**Methods:**

In a study with 87 subjects we simulated near-fall events on a perturbation treadmill and recorded them with inertial measurement units (IMU) at seven different positions. We investigated different machine learning models for the near-fall detection including support vector machines, AdaBoost, convolutional neural networks, and bidirectional long short-term memory networks. Additionally, we analyzed the influence of the sensor position on the classification results.

**Results:**

The best results showed a DeepConvLSTM with an F1 score of 0.954 (precision 0.969, recall 0.942) at the sensor position “left wrist.”

**Discussion:**

Since these results were obtained in the laboratory, the next step is to evaluate the suitability of the classifiers in the field.

## 1. Introduction

Falls are a major cause of emergency department visits in older adults (1). Approximately 33% of community-dwelling older adults fall at least once a year, with many becoming repeated fallers (2, 3). Falls reduce quality of life and increase mortality and morbidity (4). About 14% of falls result in fractures and about 10% of falls result in traumatic head injury (5, 6). Near-falls, which describe gait disturbances such as stumbling or slipping without actually falling, are frequent in everyday life and may precede actual falls. Therefore, it is important to identify near-falls, as these may present one of the earliest signs of an increased fall risk (7) providing the opportunity to establish treatment approaches as early as possible.

There is already plenty of work in the literature on fall detection. Most of the work focuses on the detection of falls and activities of daily living. According to a systematic literature review (8), wearable devices, depth-imaging cameras and radar-based systems are mainly used for fall detection. Wearable devices include smartphones with built-in accelerometers and gyroscopes. The authors describe that each of these methods has advantages and disadvantages in terms of effectiveness, data collection, security and privacy. In particular, camera-based solutions are rarely used due to privacy concerns. The first promising methods for fall detection were the threshold-based methods (9). However, it is difficult to determine the appropriate threshold without triggering false alarms: More complex movements with high outliers in the sensor signals, such as climbing stairs, are difficult to distinguish from falls or near-falls. Especially, the detection of near-fall events (stumbling) is difficult with this method, as no clear thresholds can be set that would not also be reached during normal walking.

Besides the threshold-based methods, another major area of research is fall detection using machine learning (ML) methods. Wang et al. (8) distinguished between methods based on feature engineering and neural networks. Feature engineering is used to compute selected time and frequency domain features from raw sensor data. However, according to Wang et al. (8), this approach requires expert knowledge to ultimately decide which features are most appropriate for the data and classification methods. The computed features can then be used as input to classifiers such as Support Vector Machines (SVM) or Decision Trees. Neural networks have the major advantage over conventional methods that they can learn to extract the features relevant for classification from the raw data. In the literature, convolutional neural networks (CNNs) and variants of recurrent neural networks (RNNs) are mainly used for fall detection (10, 11). Many studies use and train more complex neural networks to achieve multi-class classifications to distinguish between different activities of daily living and fall types. Also a deep convolutional and LSTM recurrent neural network (DeepConvLSTM) was used, which combines the advantages of CNNs for feature extraction and LSTM neurons for pattern recognition from the temporal sequence of the data (12). Near-falls (such as tripping) are characterized by the fact that they are intercepted by one’s own physical reaction and thus do not result in a fall. The early detection of an increased occurrence of near-falls can help to identify groups that are at risk of falls. However, the detection of near-falls has been studied by only a few research groups (13, 14), mainly due to the lack of appropriate data sets. Therefore, we provoked near-falls in older people on a perturbation treadmill in a study and measured their reaction during near-falls with inertial measurement units. Our goal was to develop a near-fall detection algorithm for early detection of increased fall risk in the future. Our research questions are:

 • Which machine learning methods are suitable for near-fall detection?
 • How does sensor position influence detection accuracy?
 • Is a single sensor sufficient for near-fall detection?

## 2. Materials and methods

### 2.1. Study design and applied sensor systems

To answer the research questions, we examined the motion sensor data from the SeFallED and the CareFall study recorded in the gait laboratory of the geriatric department of the Carl von Ossietzky University Oldenburg. The SeFallED study aims to identify long-term trajectories of older adults, presenting to the emergency department without hospital admission after a sentinel fall. For the purpose of this study, data from baseline tests were used. A full list of inclusion and exclusion criteria has been published elsewhere (15). The CareFall study investigates cardiorespiratory fitness as a potential determinant of increased fall risk and reduced gait safety in older adults. Both studies use the perturbation treadmill to induce near-falls. The experimental setup, sensors, sensor positions and orientations, and the perturbation protocols are identical. Therefore, the data of these studies are comparable and the main difference is the medical objective of the studies

**Figure 1:**
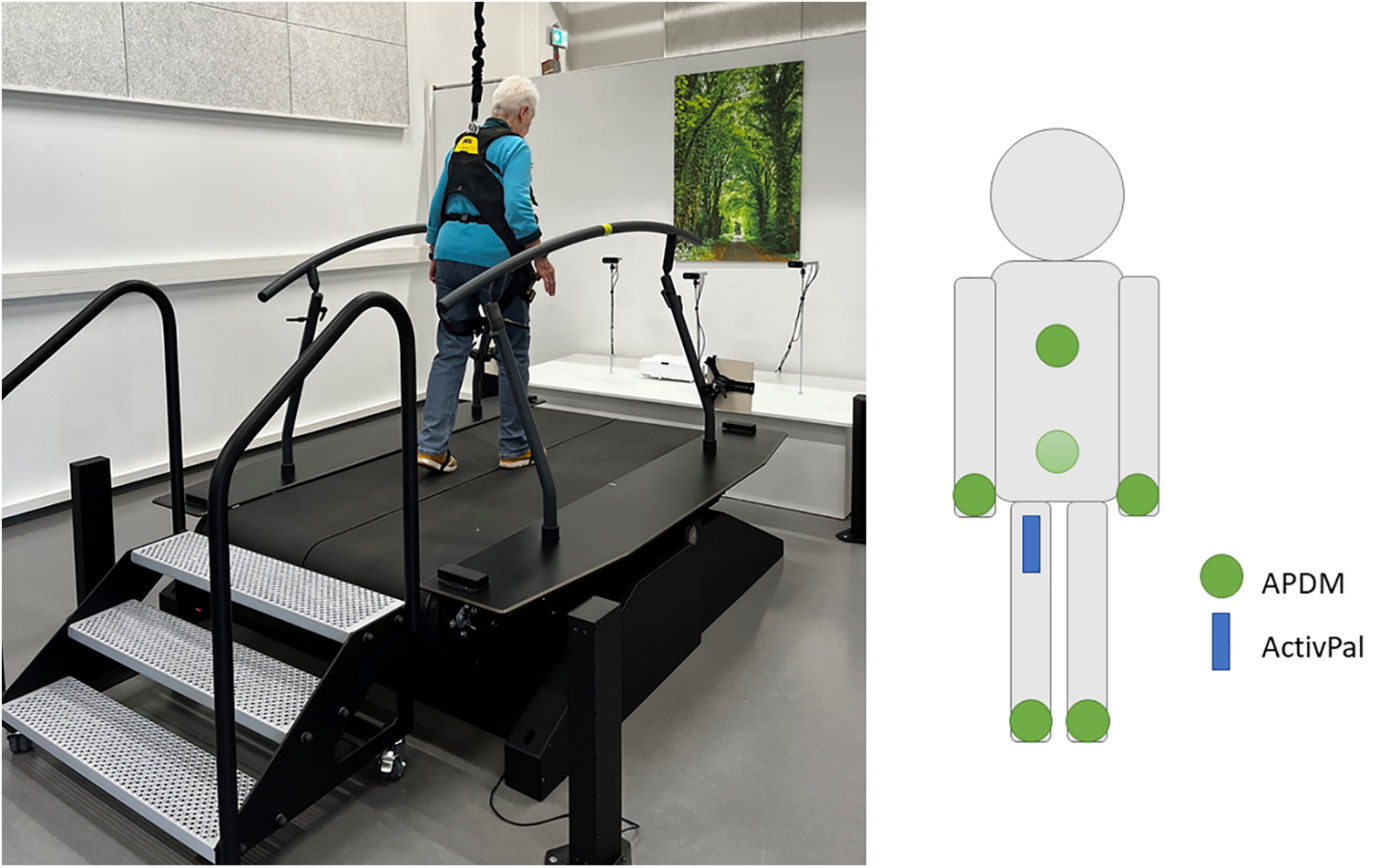
Perturbation treadmill (left) and IMU sensors and their position (right). The positions of the APDM sensors (green circle) are foot (left/right), wrist(left/right), sternum, and lumbar. The position of the activPAL sensor (blue box) is the right upper thigh.

The perturbation treadmill (Motek Medical B.V., Amsterdam, the Netherlands) induces a total of nine different disturbances in addition to the normal gait mode (see Figure 1). The perturbations include anterior–posterior, medio-lateral and pitch perturbations. The integrated split-belt of the treadmill with separate force plates (sampling rate: 300 Hz) allows to perturb either both legs or just one leg during walking. The perturbations were designed to simulate typical fall situations and to measure the subjects’ reactions. During the gait recordings, the subjects were secured with a harness to prevent them from falling to the ground. Motion sensor data was collected in both studies by six wirelessly synchronized inertial measurement units (IMU) (Opal V1, Mobility Lab™, APDM, Inc., Portland, OR, USA) and one activPAL sensor (activPAL©, PAL Technologies Ltd, Glasgow UK). The APDM Opal sensors record at a sampling rate of 128 Hz and contain an accelerometer and a gyroscope. The activPAL sensor has an accelerometer that samples at 40 Hz. Figure 1 shows the positioning of the sensors during the measurements. The sensor placements were selected based on commonly used positions in the literature (8). The sensors are always attached at the same position with the same orientation. The activPAL sensor has a long battery life is additionally worn in everyday life for a week after the laboratory visit. In the future, we also want to detect near-falls and thus an increased risk of falling with this sensor in everyday life. In addition to the IMUs, the subjects were also recorded by depth cameras (Azure Kinect DK, Microsoft, Redmond, WA, USA) during the measurements. Once the sensors were in place, first the individual preferred overground and treadmill gait speed were determined. All participants then completed a perturbation protocol with a duration of about 4 min and 30 s. The perturbation protocol starts with 30 s of the preferred treadmill walking speed, followed by 9 perturbations randomly presented to the participants. Time between perturbations will vary between 20–30 s. Additional functional assessments such as the short physical performance battery (SPPB), Short Falls Efficacy Scale International (Short-FES-I), and maximal grip strength of the dominant hand were performed. The test procedures were approved by the local ethics committee (ethical vote: Carl von Ossietzky University Oldenburg No. 2021-120 and 2021-026) and conducted in accordance with the Declaration of Helsinki. In total the data of 87 participants (44 male, 43 female) were included in this work. All participants provided written informed consent to participate in the study. Table 1 shows the characteristics of the study population. Of the 87 subjects, eleven reported the use walking aids in their daily lives and 42 reported no treadmill-experiences at all.

**Table 1:** Characteristics of the entire study population with mean, median, standard deviation (SD), minimum (min), maximum (max), 25th percentile (Q1) and 75th percentile (Q3) values.

	N	Mean	Median	SD	Min	Max	Q1	Q3
Age [years]	87	73.5	73.0	6.4	62.0	87.0	68.0	79.0
Height [cm]	87	171.3	172.0	10.6	145.0	192.0	162.0	179.0
Body mass [kg]	87	77.0	75.4	14.1	43.9	119.7	67.2	87.0
BMI [kg m${}^{-2}$]	87	26.2	25.3	4.2	19.6	42.7	23.4	27.8
SPPB [score]	84	10.5	11.0	1.8	4.0	12.0	10.0	12.0
Short-FES-I [score]	87	8.8	8.0	2.5	7.0	20.0	7.0	10.0
Number of diagnoses	87	3.1	3.0	2.2	0.0	10.0	1.0	4.0
Max grip strength [kg]	86	32.8	32.1	10.6	10.0	66.1	24.4	40.3
GS overground [km h${}^{-1}$]	87	4.4	4.5	0.9	1.8	6.4	3.9	5.0
GS treadmill [km h${}^{-1}$]	87	3.5	3.6	0.9	0.8	5.7	3.0	4.2
GS treadmill to overground [%]	87	78.6	80.0	12.7	30.0	108.0	70.0	88.0

BMI, body mass index; SPPB, short physical performance battery; Short-FES-I, short falls efficacy scale international; GS, preferred gait speed.

**Figure 2:**
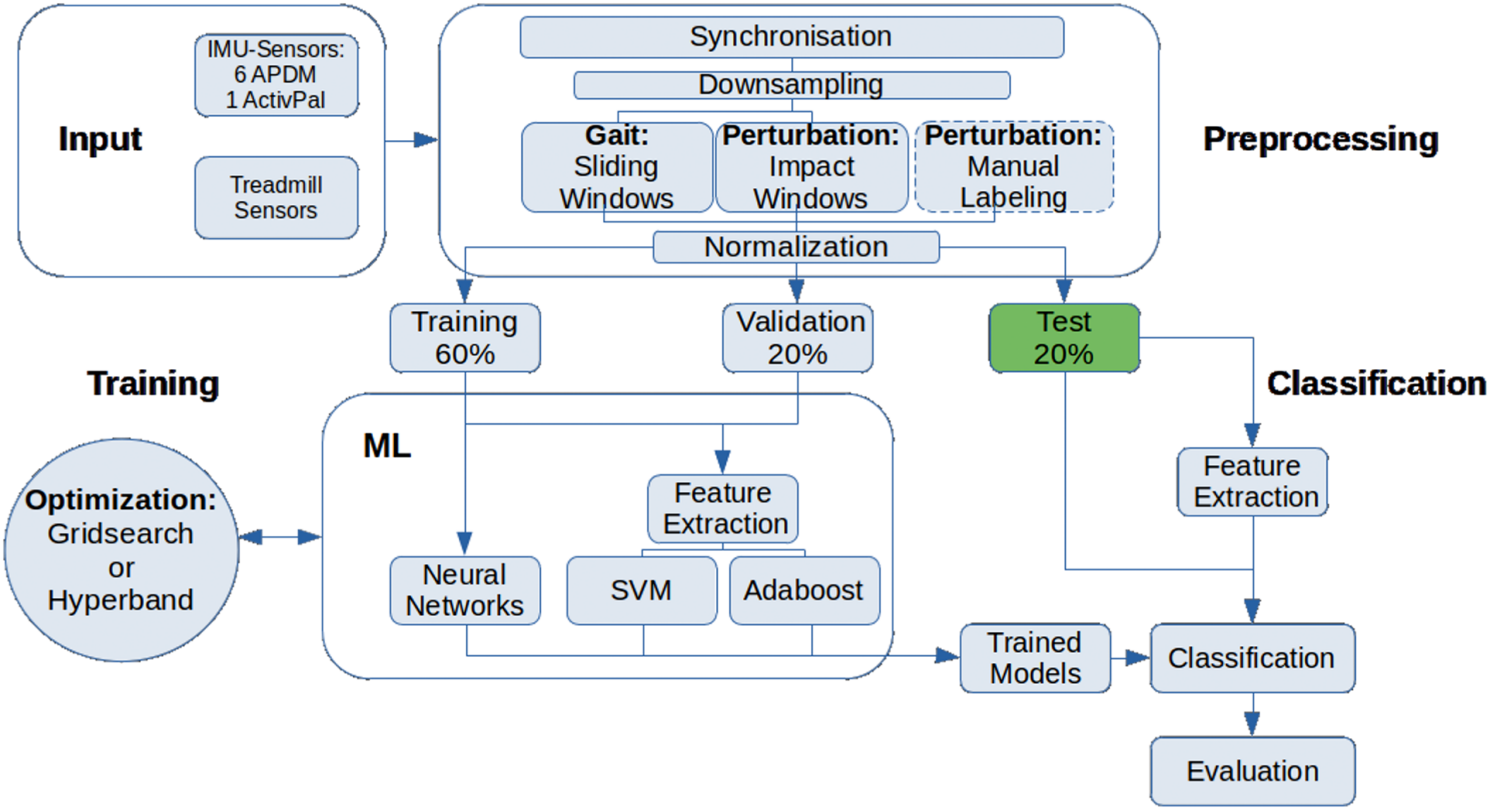
Overview of the methods used to preprocess, train and classify the sensor datasets.

After data acquisition, the sensor data were processed as shown in Figure 2. The processing includes preprocessing steps, training, and classification, which were described in the following section. We focused on acceleration data only for all analyses.

### 2.2. Data preprocessing

The treadmill and the IMU-computers, both APDM and activPAL, are synchronized using Network Time Protocol. The treadmill and APDM are also synchronized by an external trigger so that these measurements start at the same time. In addition to NTP, we use event identification to synchronize the activPAL sensor (change between static and dynamic activity, response to disturbance). For synchronization, the data from both IMU-sensors (activPAL and APDM) was adjusted to the sampling rate of the treadmill. Subsequently, the data was labeled automatically, distinguishing between walking and perturbations. The classification was based on the treadmill data, which included timestamp and duration of the perturbations. All gait recordings without perturbations are directly classified into 2 s windows using the sliding window method. The duration of the perturbation does not necessarily represent the response to the perturbation, as individual response times must be considered. Thus, the perturbation windows are determined using the following methodology. Based on the time window of the perturbation, the method of Liu et al. (16) is used to calculate the maximum value of the norm from the three axes of the sensors using the formula (1).

(1) $$\max_{norm}=\max(\sqrt{x^{2}+y^{2}+z^{2}})$$

**Figure 3:**
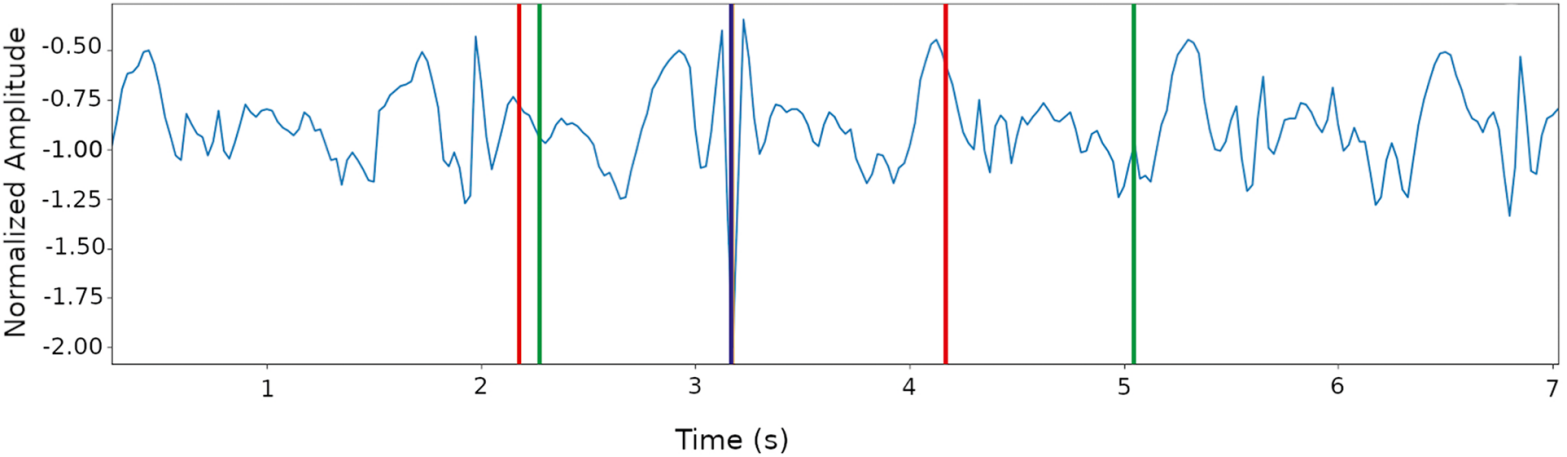
Creating a perturbation window around the maximum of the norm, which represents the maximum reaction to a perturbation. Green: Treadmill perturbation, blue: Maximum, red: Selected 2s perturbation window.

The maximum is assumed to be the center of the perturbation response. Then a 2 s window is cut around the maximum. Figure 3 shows such an impact window. In the area between the two green lines, the treadmill has triggered a perturbation. The found maximum of the norm (negative peak) is marked with the blue line. The cut window is the area between the two red lines. This procedure must be repeated for each sensor position since the position of the respective maximum and thus the reaction time of the extremities can be different. The windows created and assigned to the two classes are then randomly divided into training, validation, and test data sets. The training data set receives 60%, the validation data set 20% and the test data set the remaining 20% of the windows. To ensure a comparable evaluation of all methods used, the division into the three data sets remains identical for all machine learning approaches.

### 2.3. Training

We used different machine learning algorithms to identify the best method for our application. The selection of the algorithms was based on the present literature in the field of fall detection (8). The classification algorithms include support vector machines (SVM) (17), AdaBoost (18), one- and two-dimensional convolutional neural networks (1D- and 2D-CNN) (19), and bidirectional long short-term memory (LSTM) networks (20). The improvement in bidirectional LSTM is that the current output is not only related to previous information but also to subsequent information and therefore captures a longer temporal relationships to enhance the recognition rate (21). For the SVM and AdaBoost classification, we selected features according to Liu et al. (22) and calculated them for each time window. For each axis of the accelerometer data (x,y,z) as well as Norm xyz, Norm xy, Norm xz following features are calculated:

 • mean, median, standard deviation
 • interquartile range, variance, minimum, maximum
 • max-min (range), kurtosis, skew, energy
 • Pearson correlation coefficient of the axes

All neural networks were created using tensorflow and keras modules. The optimization of the hyperparameters was done with hyperband for the neural networks and GridSearchCV for SVM and AdaBoost. Table 2 shows the search parameters for GridSearchCV and Hyperband. To compensate for the imbalance of classes (6238 gait windows and 1107 perturbation windows), we used weighted classes (23) as well as data augmentation (24).

**Table 2:** Search parameters for GridSearchCV und Hyperband.

Model	Search method	Investigated parameters
SVM	GridSearchCV	kernel (linear, rbf, poly)
		C (0.01, 0.1, 1.0, 10.0)
		gamma (1, 0.1, 0.01, 0.001)
		degree (2, 3, 5, 7)
		Data augmentation (True, False)
		Class weights (True, False)
AdaBoost	GridSearchCV	max_depth (2, 4, 6, 8, 10)
		min_samples leaf (2, 5, 10)
		n_estimators (10 , 25, 50, 100, 250)
		learn_rate (0.001, 0.01, 0.1, 1)
		Data augmentation (True, False)
		Class weights (True, False)
1D-CNN/CNN	Hyperband	Hidden_Layer 1-3 (32, 64, 96, 128, 160, 192, 224, 256)
		kernel_size (3, 5, 7)/(3×3, 5×5, 7×7)
		L2_kernel_regularization (True, False)
		learn_rate (0.0001, 0.001, 0.01)
		batch_size (64, 128, 256)
		Data augmentation (True, False)
		Class weights (True, False)
DeepConvLSTM	Hyperband	CNN_Layer 1-4 (32, 64, 128, 256)
		LSTM_Layer 1-2 (32, 64, 128, 256)
		L2_kernel_regularization (True, False)
		Learnrate (0.0001, 0.001, 0.01)
		Batchsize (64, 128, 256)
		Data augmentation (True, False)
		Class weights (True, False)

## 3. Results

To answer our research question “Which machine learning methods are suitable for near-fall detection?,” we divided our results into classical ML approaches (SVM and AdaBoost) and neural networks. The results are also sorted by sensor position to answer the question “How does sensor position affect detection accuracy? Finally, we investigate the question” Is a single sensor sufficient for near-fall detection? To do this, we examine whether the combination of all six APDM sensors provides better results than a single sensor.

### 3.1. SVM and AdaBoost

For each sensor and sensor position, the GridSearch procedure was used to find the best classifier with the optimal hyperparameters in each case. For AdaBoost, GridSearch showed for example that DecisionTrees with max_depth=8 and min_samples_leaf=10 should be used as weak learners. It also showed that a total of 250 weak learners should be used. Table 3 shows the GridSearch results for SVM for each sensor position. The results of the different sensor positions of the SVMs and the AdaBoost classifier are presented in Table 4.

**Table 3:** Results of the GridSearch with SVM for each sensor position.

Sensor position	C	Degree	gamma	kernel
activPAL (upper thigh)	1			linear
APDM (sternum)	0.1			linear
APDM (lumbar)	1		0.001	rbf
APDM (wrist right)	10	3	0.01	poly
APDM (wrist left)	10			linear
APDM (foot right)	0.1	2	0.01	poly
APDM (foot left)	0.1	5	0.01	poly

**Table 4:** Classification results of the different sensor positions with the SVM classifier and AdaBoost.

Sensor position	Classifier	Precision	Recall	F1	Training duration (s)
activPAL (upper thigh)	SVM	0.947	**0.790**	**0.862**	<2
	AdaBoost	**0.952**	0.784	0.860	32
APDM (sternum)	SVM	0.240	0.748	0.364	<2
	AdaBoost	0.200	0.685	0.310	30
APDM (lumbar)	SVM	0.390	0.688	0.498	<2
	AdaBoost	0.553	0.696	0.616	30
APDM (wrist right)	SVM	0.579	**0.860**	0.692	<2
	AdaBoost	**0.707**	0.737	**0.722**	31
APDM (wrist left)	SVM	0.560	0.734	0.635	<2
	AdaBoost	0.599	0.664	0.630	31
APDM (foot right)	SVM	0.254	0.842	0.391	<2
	AdaBoost	0.269	0.828	0.406	34
APDM (foot left)	SVM	0.411	0.783	0.539	<2
	AdaBoost	0.338	0.765	0.469	34

The best results for activPAL and APDM sensor are written in bold.

Data augmentation did not lead to any significant improvement in results and is therefore not listed here. The results show that we achieved similar results for SVM and AdaBoost for the activPAL sensor on the “upper thigh.” Adaboost achieved a precision of 0.952 and SVM a recall of 0.790 and an F1-Score of 0.862. For the APDM sensor the sensor position “wrist right” achieved the best results for SVM and AdaBoost with a precision of 0.707 (AdaBoost), a recall of 0.860 (SVM), and an F1-Score of 0.722 (AdaBoost).

### 3.2. Neural network

For each sensor and sensor position, the optimized hyperparameters for each evaluated network architecture was determined. Table 5 lists the results for the hyperparameters.

**Table 5:** Optimized hyperparameters for each evaluated network architecture.

Parameter	1D_CNN	CNN	DeepConvLSTM	Bi-LSTM
Class weights	Yes	Yes	Yes	Yes
Augmentation	Yes	No	No	No
Overlap	No	No	No	No
L2	Yes	No	No	No
Hidden layer 1	96 (Conv1D)	96 (Conv2D)	64 (4 × Conv2D)	128 (Bi-LSTM)
Hidden layer 2	128 (Conv1D)	128 (Conv2D)	128 (2 × LSTM)	64 (Bi-LSTM)
Hidden layer 3	64 (Conv1D)	32 (Conv2D)		32 (Dense)
Kernel size	5	(5×5)	(5×1)	
Batchsize	64	128	128	64
Learnrate	0.001	0.001	0.001	0.001
Dropout	0.2	0.2	0.2	0.2
Trainable parameters (activPAL)	145,793	433,377	948,545	303,681
Trainable parameters (APDM)	227,713	474,337	2,259,265	303,681

For the following analyses, we calculated the test results as the mean of 10 test runs and the standard deviation for each metric. Table 6 shows the results for each network architecture and sensor position. All data sets were tested with and without data augmentation (DA). The best result is listed. While the bidirectional Long Short-Term Memory (Bi-LSTM) networks produced weaker results for the precision and correspondingly also for the F1-Score, good results were obtained for each of the other architectures. For the activPAL sensor with the position upper thigh the 1D-CNN with DA achieved the best results with a precision of 0.838, a recall of 0.867 and an F1-Score of 0.851. For the APDM sensor, the left wrist shows the best precision of 0.969 with DeepConvLSTM + DA. The best recall of 0.974 was achieved with the CNN and the left foot as sensor position. The best F1-Score was 0.954 with DeepConvLSTM + DA on the left wrist.

**Table 6:** Comparison of the test results for all sensor positions and classification architectures.

Sensor position	Architecture	Precision	Recall	F1
activPAL (upper thigh)	Bi-LSTM	0.549 ± 0.042	0.860 ± 0.061	0.669 ± 0.044
	DeepConvLSTM	0.743 ± 0.097	0.814 ± 0.058	0.773 ± 0.063
	CNN + DA	0.749 ± 0.112	0.837 ± 0.043	0.785 ± 0.072
	1D-CNN + DA	**0.838** ± 0.081	**0.867** ± 0.042	**0.851** ± 0.054
APDM (sternum)	Bi-LSTM	0.474 ± 0.121	0.755 ± 0.130	0.563 ± 0.071
	DeepConvLSTM	0.803 ± 0.082	0.938 ± 0.025	0.862 ± 0.045
	CNN + DA	0.768 ± 0.117	0.897 ± 0.043	0.823 ± 0.077
	1D-CNN	**0.840** ± 0.079	**0.943** ± 0.018	**0.886** ± 0.042
APDM (lumbar)	Bi-LSTM	0.496 ± 0.139	0.848 ± 0.062	0.613 ± 0.118
	DeepConvLSTM + DA	**0.872** ± 0.050	0.934 ± 0.027	0.900 ± 0.021
	CNN + DA	0.863 ± 0.084	0.959 ± 0.335	**0.904** ± 0.040
	1D-CNN	0.829 ± 0.040	**0.963** ± 0.025	0.890 ± 0.020
APDM (wrist right)	Bi-LSTM + DA	0.715 ± 0.143	0.837 ± 0.056	0.765 ± 0.096
	DeepConvLSTM + DA	**0.956** ± 0.026	0.845 ± 0.040	0.896 ± 0.014
	CNN + DA	0.913 ± 0.053	**0.935** ± 0.029	**0.922** ± 0.028
	1D-CNN	0.902 ± 0.042	0.882 ± 0.044	0.891 ± 0.031
APDM (wrist left)	Bi-LSTM	0.728 ± 0.111	0.929 ± 0.024	0.810 ± 0.068
	DeepConvLSTM + DA	**0.969** ± 0.011	0.939 ± 0.192	**0.954** ± 0.010
	CNN	0.959 ± 0.028	0.935 ± 0.026	0.946 ± 0.014
	1D-CNN + DA	0.951 ± 0.026	**0.942** ± 0.021	0.946 ± 0.013
APDM (foot right)	Bi-LSTM	0.583 ± 0.094	0.754 ± 0.072	0.651 ± 0.067
	DeepConvLSTM	0.873 ± 0.077	0.941 ± 0.028	0.904 ± 0.049
	CNN	**0.947** ± 0.041	0.874 ± 0.103	0.906 ± 0.068
	1D-CNN	0.920 ± 0.047	**0.950** ± 0.027	**0.934** ± 0.025
APDM (foot left)	Bi-LSTM	0.744 ± 0.037	0.888 ± 0.061	0.808 ± 0.024
	DeepConvLSTM	0.925 ± 0.047	0.944 ± 0.034	0.933 ± 0.025
	CNN	0.926 ± 0.017	**0.974** ± 0.016	**0.949** ± 0.009
	1D-CNN	**0.938** ± 0.026	0.902 ± 0.064	0.918 ± 0.035

The test results are listed as the mean of 10 test runs ± standard deviation. The best results for each sensor position are written in bold type.

### 3.3. Single sensor vs. multi-sensor system

**Figure 4:**
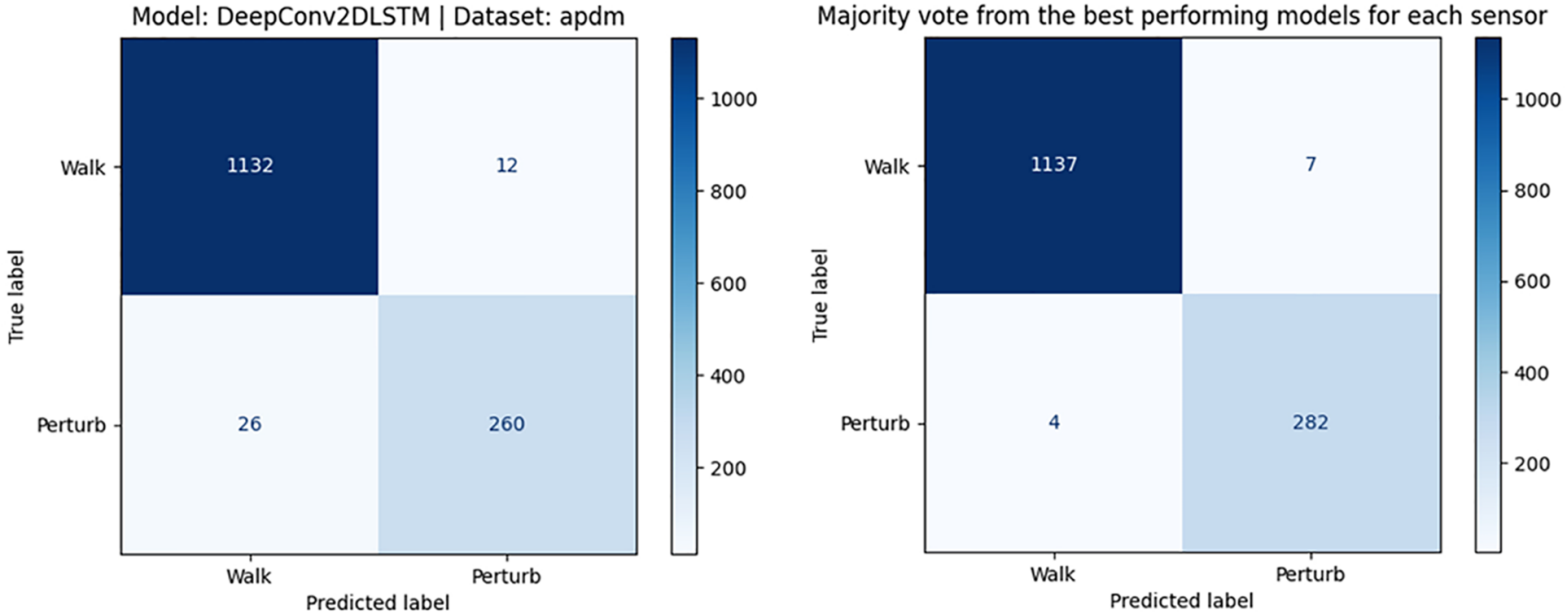
Confusion matrix of DeepConv2DLSTM model and optimal hyperparameters based on data of a multi-sensor system (left) and confusion matrix of the majority vote classification of a multi-sensor system (right).

We also examined whether the combination of all six APDM sensors provides better results than a single sensor. Therefore, we realized two approaches. Firstly, we trained the models on all APDM acceleration data, and therefore the combination of all sensor positions. Secondly, we used the best classifier for each sensor position (Table 6) and developed a majority vote classification algorithm. So the class with the majority of the six single votes is selected. The confusion matrices are presented in Figure 4.

For the first approach, the combination of all sensor positions, best results are obtained with the DeepConv2DLSTM model and optimized hyperparameters. The F1-Score was of 0.932, precision 0.956, and recall 0.909. The majority vote classification approach resulted in an F1-Score of 0.995, precision of 0.994 and a recall of 0.996. There was no significant difference in classification time between these approaches when using pre-processed data.

## 4. Discussion

### 4.1. Influence of machine learning method

In this work, machine learning methods were used to classify near-fall events in motion sensor data. For this purpose, the suitability of different machine learning methods was evaluated. We included feature-based ML classifiers like SVM and AdaBoost as well as neural networks like 1D- and 2D-CNN, and LSTM networks. For the feature-based classifiers, we obtained good results for the activPAL sensor on the right upper thigh (Table 4). Here, the SVM classifier achieves an F1 score of 0.862 and the Adaboost classifier achieves an F1 score of 0.860. The precision and recall values of both classifiers are also close to each other. For both classifiers, the achieved precision is relatively high (>0.9), while the recall is significantly lower (<0.8). The low recall shows that a higher number of perturbations are falsely classified as gait data (false positives). To improve the results in the future, we may test more features in combination with the features already used. These could be, for example, features from the frequency domain, which are also widely used in the literature (25, 26).

The classification of the data was also investigated using neural networks (Table 6). While the bidirectional Long Short-Term Memory networks produced weak results for the precision, better results were obtained for each of the other architectures. The best architecture for the activPAL is the 1D-CNN+DA with an F1 score of 0.851. Another important point to compare is the training and testing time. To compare the resources required, it is assumed that the data are already prepared for the respective classifier. Training and testing the SVM classifier takes less than 2 s. Training with the 1D-CNN requires a graphics card. This training takes an average of 13.9 s without data augmentation and 25.1 s with data augmentation. After the model is trained once, it can be loaded for further classifications of test data without a new training. Loading the saved model and testing it with new data then takes about 3 s. For comparison of the evaluated methods it is also important to consider how they can be implemented for the actual use for the early detection of fall risk in private homes. Depending on the available hardware the size of the neural networks may be a factor in deciding which method should be used. From the comparison of the tested methods, it can be seen that the DeepConvLSTM model achieves the best result when the focus is on the correct classification of perturbations with few false positives (high precision). The DeepConvLSTM model however requires the most hardware resources of all evaluated methods. When less hardware resources are available, the SVM classifier can also give good results. When using the SVM classifier, it must then be weighed how to handle the higher number of false positives.

### 4.2. Influence of sensor position

We also investigated the influence of the sensor position on the classification results. Especially, we wanted to examine whether an alternative sensor position is better for classification compared to the positioning on the thigh, which is used in our studies for the activPAL sensors to detect near-falls in everyday life. Compared to the results with the activPAL sensor data, the classifications with the APDM sensors and the SVM and Adaboost classifiers achieve worse F1-Scores (Table 4). The APDM sensors on the right wrist achieves the best F1-Score among the APDM sensors with 0.722. But, it is noticeable that the precision is significantly smaller for all classifiers with the APDM data than in the experiments with the activPAL data. The classifiers that only achieve a small precision value can therefore not distinguish well between gait data without perturbations and those with perturbations and would thus trigger many false alarms. The best results for the neural networks (Table 6) were obtained with a DeepConvLSTM model on the left wrist with an F1-Score of 0.954 as well as a CNN on the data of the left foot with an F1-Score of 0.949. This work shows that the sensor position influences the classification. We achieved the best results with the sensor position “left wrist.” But F1-Scores above 0.85 could also be obtained with all other positions and they are therefore also suitable. Our dataset was created under laboratory conditions. Disturbances were provoked externally by the treadmill, but the subjects still knew they were going to stumble at some point and were therefore able to prepare for it. Thus, the best sensor position in real life may differ from laboratory conditions.

### 4.3. Influence of sensor number

We also investigated if a multi-sensor system achieved better results than a single sensor and analyzed two approaches: training on a combined data-set of all six APDM sensors and a majority vote classification based on the best classifier for each APDM sensor. As already discussed, satisfactory results could be reached by using only the accelerometer data of one sensor. For the trained classifier with data from all APDM sensors, we found that the best model was the DeepConvLSTM with an F1-Score of 0.932. For the majority vote classification, the F1-Score is 0.995. Thus, the multi-sensor system with majority vote performs slightly better than a single sensor (F1-Score 0.954). However, it is also important to note that a near-fall detection system for older adults that requires wearing six sensors is not suitable for everyday use.

### 4.4. Potential for fall prevention

As the data were recorded under laboratory conditions with participants being equipped with a total of 7 sensors, transfer of the results into everyday life is limited. Nevertheless, the results are promising that sensor-based near fall detection may be possible in the future warranting further research to design translational methods. In the next step, we will analyze the activPAL data recorded over one week in the participants’ everyday life. Here we will work with a hierarchical classifier to first identify the gait phases and then use the algorithm presented in this article to search for near-falls in these gait phases. The participants kept a near-fall/fall log. This allows us to evaluate our results. In this context, the similarity between real and treadmill induced near-falls should be investigated.

However, if it was possible to validly identify individuals at a high risk for falls by them wearing a sensor on their thigh, it would provide the opportunity to refine preventive treatment approaches by identifying individuals at risk as early as possible. Early identification is crucial to establish effective falls prevention (27) and, thus, avoid a fall-and all the associated negative outcomes-itself. The potential of e-health, technology and sensor-based data in falls prevention has been acknowledged in the world guidelines for falls prevention published in 2022 (28). However, the guidelines further support the notion that more research is needed to fully unlock the potential of such technology-based approaches.

## 5. Conclusion

Early detection of a fall risk is important in order to initiate interventions. We developed an algorithm to detect near-falls and investigated the suitability of different machine learning methods. We obtained the best results with a DeepConvLSTM based on data of a single sensor on the left wrist. However, promising results were also reached at other sensor positions. A multi-sensor system with majority vote performs slightly better than a single sensor. Since these results were obtained in the laboratory, the next step is to evaluate the suitability of the classifiers and sensor position in the field.

## 6. Outlook

As an outlook, our system can also be adapted to a fall detection system with an alarm function. Therefore, the dataset and the machine learning algorithms need to be extended in an appropriate way. Transfer learning could be a relevant approach for this adaptation (29, 30). Another important use case for transfer learning could be the individualization of the machine learning model for people with specific movement patterns (due to certain diseases or disabilities) to reduce the rate of false positive alarms. More advanced machine learning methods, such as graph neural networks (31) and evolutionary analysis (32), should also be considered for future work, since they could improve the performance of the (near-)fall detection systems. Another commonly used technique in modern machine learning research is the attention mechanism. Li et al. (33) implemented an improved attention mechanism together with a CNN-BiLSTM network to further identify important regions in the data. An implementation of this method in combination with the already used DeepConvLSTM network could be an improvement to our current approach. A fall detection system also requires the implementation of a continuous recording and analysis of the motion data. This could enable the proposed system to react to falls in real time and raise an alarm for potential emergencies. Many systems already have a long battery life of for example one week, but even recharging once a week can be a challenge for this group of older people at high risk of falling. For continuous measurement systems, a self-powered sensor could therefore be an interesting development to reduce data loss due to battery life (34). Developments regarding multi-sensor systems are also interesting to record and link for example vital parameters such as breathing patterns in addition to activity data to identify emergency situations (35, 36).

## Data Availability

The datasets presented in this article are not readily available because the raw data of the studies contain sensitive health data and cannot be made available due to data protection regulations. Requests to access the datasets should be directed to sandra.hellmers@uol.de.
